# Books and Pamphlets Received

**Published:** 1887-08

**Authors:** 


### BOOKS AND PAMPHLETS RECEIVED.

A pamphlet treating on the eminent medicinal properties of Sander & Sons' Eucalypti Extract (Eucalyptol). The little book, supplied gratis on application to Dr. Sander, Dillon, Iowa, illustrates, in a succinct form, the splendid results obtained at the clinics of the Universities of Bonn and Greifswald, and shows beyond question that the genuine drug governs, in its effects, a very varied class of complaints. The details given are very instructive, and must prove of great advantage to every practitioner.

Tracts on Massage. No. III. The Uses of Massage in Medical Practice. Translated from the German of Reibmayr, with notes, by Benjamin Lee, A. M., M.D., Ph D., Secretary of the State Board of Health of Pennsylvania, late President of the American Academy of Medicine, member of the American Medical Association, etc.

Practical Examples in Prescription Writing. By Charles H. May, M.D. Issued for the use of his quiz classes.

Diana; a Psycho-Fyziological Essay on Sexual Relations, for Married Men and Women. Third edition. Revised and enlarged. New York, Burnz & Company, 24 Clinton Place. 1885.

Infants, their Chronological Progress. By Prof. Standford E. Chaille, M. D., Tulane University of Louisiana.

Some Considerations Concerning Cancer of the Uterus, Especially its Palliative Treatment in its Later Stages. By Andrew F. Currier, M.D. Reprinted from the *New York Medical Journal* for March 5, 1887.

Resident Students of the Charity Hospital of New Orleans, Louisiana. Establishment of a Medical Library, by the Louisiana State Medical Society. By Joseph Jones, M.D., President of the Louisiana State Medical Society, Visiting Physician of the Charity Hospital of New Orleans, Professor of Chemistry in the Medical Department of Tulane University of Louisiana.

Researches into the Etiology of Dengue. By J. W. McLaughlin, M.D., of Austin, Texas.

The Relation of the Nervous System to Hæmophilia, Malarial Hæmaturia, Etc. Second paper. By C. H. Hughes, M.D., St. Louis. Reprint from *The Alienist and Neurologist*, July, 1887.

A Unique Case of Bi Lateral Athetosis. By C. H. Hughes, M.D., St. Louis, Neurologist on the Staff of St. Louis Protestant Hospital, Lecturer on Nervous Diseases, St. Louis Medical College, etc. Reprint from *The Alienist and Neurologist*, St. Louis, July, 1887.

Medicine and Medicine-Men. Anniversary address delivered at the banquet of the Louisville Medical Society, May 26, 1887. By John Godfrey, Surgeon. M.-H. S. Louisville, Ky. John P. Morton & Co., printers. 1887.

To the Medical Profession. Dr. Dudley S. Reynolds, of Louisville, Kentucky—His Title to the Censorship of the Medical Profession. A biographical sketch.

Transplantation of a Rabbit's Eye into the Human Orbit. By Charles H. May, M.D. Reprinted from the *Archives of Ophthalmology*, Vol xvi., No. 1, 1887.

*Anthem Treasures*, a collection of Choice Anthems, both new and old, for all occasions of public worship, and a large and complete department of funeral lyres. By J. M. Stillman, Mus. Doc., and S. W. Straub, Chicago. Published by S. W. Straub.

We have here a large work of over 300 pages, containing a great number of choice and beautiful anthems, new and old. They are specially adapted to choirs, to celebrations, soirees or other places where fine displays of musical talent are expected. They may be had of S. W. Straub, Chicago, at $1.25 per copy, or $12 per dozen.

I. PHILLIPS.—The Surgical Instrument House of Atlanta. See his new and interesting advertisement in this journal.

SEABURY & JOHNSON.—Read the advertisement from the above excellent house, entitled Caution to Physicians. It contains matter of special interest to practitioners.

JEFFERSON MEDICAL COLLEGE.—Read the advertisement of the Jefferson Medical College in this issue.

SOUTHERN MEDICAL COLLEGE.—See the advertisement of the Medical and Dental Departments of the Southern Medical College, Atlanta.

